# Emerging innovative immunotherapies in malignant ascites: current landscape, challenges and perspectives

**DOI:** 10.3389/fonc.2026.1893803

**Published:** 2026-10-02

**Authors:** Xian Liu, Xiaobo Peng, Xiaoxiao Wu, Yue Wu, Tong Yin, Jingyu Liu, Huawei Xu, Xianbao Zhan

**Affiliations:** Department of Oncology, Changhai Hospital, Naval Medical University, Shanghai, China

**Keywords:** malignant ascites, immune checkpoint inhibitors, T-cell engagers, oncolytic viruses, cancer vaccines

## Abstract

Malignant ascites is a common, severe complication of advanced solid tumors, driven by multifactorial pathogenesis, including impaired lymphatic drainage, vascular endothelial growth factor-induced vascular hyperpermeability, pro-permeability inflammatory cytokines, and profound peritoneal immunosuppression. Clinically, it correlates with poor prognosis, reduced overall survival, and substantial quality-of-life deterioration. Current treatment remains predominantly palliative: repeated large-volume paracentesis offers transient symptom relief, fails to prevent reaccumulation, and carries procedural risks. Emerging evidence indicates malignant ascites is not merely passive fluid accumulation but an active, immunosuppressive niche shaped by bidirectional crosstalk among tumor cells, stromal components, diverse immune subsets (e.g., regulatory T cells, myeloid-derived suppressor cells, dysfunctional CD8^+^ T cells), and soluble mediators. This narrative review summarizes recent advances in immunomodulatory treatment for malignant ascites, with an emphasis on strategies targeting the ascitic immune microenvironment. Intraperitoneal administration of immune checkpoint inhibitors has shown acceptable safety and preliminary activity in early-phase single-arm trials, although confirmatory data from larger controlled studies are lacking. In randomized trials, the T-cell engager catumaxomab prolonged puncture-free survival, while the bispecific antibody M701 has shown similar benefits in a phase II randomized controlled study; these findings support further investigation of localized T-cell redirection. Preclinical and early-phase clinical studies suggest that intraperitoneal oncolytic viruses can induce immunogenic tumor cell death and T-cell activation, with single-cell analyses indicating remodeling of the ascitic immune microenvironment. Future directions include integrating ascites immune phenotyping, patient-derived organoids for drug sensitivity testing, and machine learning-based decision support to guide personalized immunotherapy.

## 1 Introduction

Malignant ascites is a common, clinically significant complication of advanced intra-abdominal solid tumors. Its pathogenesis is multifactorial, involving tumor-mediated lymphatic obstruction, vascular endothelial growth factor (VEGF)-driven peritoneal microvascular hyperpermeability, and pro-inflammatory cytokine (e.g., IL-6, IL-8)-mediated endothelial barrier disruption within the peritoneal microenvironment (1). Clinically, its presence strongly suggests peritoneal carcinomatosis and portends a substantially worse prognosis, most frequently in gastric, colorectal, pancreatic, and gynecologic malignancies. In pancreatic ductal adenocarcinoma (PDAC), malignant ascites is an independent predictor of poor survival, with a hazard ratio (HR) for mortality of 2.03 (95% CI: 1.47-2.81)—greater than that associated with liver or lung metastases (2). Similarly, patients with advanced gastric or colorectal cancer and concomitant peritoneal metastasis plus ascites exhibit markedly reduced median overall survival compared with those without ascites, as well as higher risks of cancer-associated malnutrition, systemic cachexia, and functional decline (e.g., Eastern Cooperative Oncology Group [ECOG] performance status ≥2) (1). Moreover, progressive ascites accumulation commonly causes debilitating symptoms, including abdominal distension, pain, early satiety, dyspnea on exertion, and anorexia, thereby impairing health-related quality of life and diminishing tolerance to systemic or locoregional therapies. In refractory cases, recurrent paracentesis and frequent hospitalizations impose substantial physical, psychological, and economic burdens on patients and healthcare systems alike (2).

Currently, clinical management of malignant ascites remains predominantly palliative. Therapeutic paracentesis is the most widely used intervention for rapid symptomatic relief—particularly for abdominal distension and dyspnea. However, it provides only transient mechanical decompression without addressing the underlying pathophysiological drivers of ascites formation. Repeated paracentesis carries well-recognized risks: post-procedural hypotension, spontaneous bacterial peritonitis, and abdominal wall leakage, all of which can exacerbate patients’ psychological distress and unduly strain healthcare resources (3, 4). Alternative approaches such as diuretics, intraperitoneal chemotherapy, and peritoneovenous shunting have been investigated, but it is important to acknowledge that robust clinical evidence is lacking, standardized treatment guidelines do not exist, and therapeutic efficacy varies substantially depending on tumor histology, peritoneal carcinomatosis burden, and patient performance status. Therefore, reliance on local, symptom-directed interventions like paracentesis is insufficient to alter disease trajectory, which makes the development of mechanistically informed, disease-modifying therapeutic strategies an urgent priority (4).

Beyond gastric and ovarian cancers, malignant ascites is also a frequent complication in colorectal and pancreatic cancers. In metastatic colorectal cancer (mCRC), the prevalence of synchronous peritoneal metastasis ranges from 4%-5% to 20% based on autopsy findings (1). In a pooled analysis of 900 mCRC patients from prospective trials, 39 (4.3%) had peritoneal metastasis with malignant ascites (1). In pancreatic ductal adenocarcinoma (PDAC), the incidence of ascites is considerably higher. A prospective cohort study of 437 PDAC patients reported that 182 (41.6%) developed ascites (5), while an analysis of 93,330 hospitalized pancreatic cancer patients found that 16.2% had concurrent ascites (6). In a retrospective study of 581 patients with metastatic pancreatic cancer, 122 (21.0%) developed ascites after a median of 8.7 months from diagnosis of metastatic disease (7). Collectively, these figures demonstrate that colorectal and pancreatic cancers, alongside gastric and ovarian cancers, represent frequent causes of malignant ascites, underscoring its broad clinical relevance across major gastrointestinal and gynecologic malignancies.

Malignant ascites is increasingly regarded not as a passive consequence of tumor progression but as a dynamic, immunologically active microenvironment made up of tumor cells, multiple immune subsets, stromal components, and numerous soluble mediators that all actively contribute to tumor progression, immune evasion, and therapeutic resistance. Therefore, it is a biologically distinct and meaningful entity. The therapeutic target has been clearly discussed in the literature (8), and there is now clinical support from the report by Chen et al. (9), who described a case of pancreatic cancer with peritoneal metastasis in which intraperitoneal administration of autologous ex vivo-expanded natural killer (NK) cells together with a PD-1 inhibitor resulted in eradication of malignant cells from the ascites, along with T-cell activation and repolarization of tumor-associated macrophages. The treatment promotes the shift toward the pro-inflammatory M1 phenotype and thereby restores anti-tumor immunity within the ascitic microenvironment. More importantly, the peritoneal cavity functions as a functionally specialized but interconnected immune “compartment” that is both anatomically distinct from the systemic circulation, yet remains immunologically connected through continuous cellular trafficking, lymphatic drainage, and cytokine exchange. Consequently, peritoneal carcinomatosis is associated with a distinct immune landscape characterized by elevated pro-inflammatory cytokines alongside a complex cellular milieu that includes abundant lymphocytes—notably clonally expanded T cells—as well as macrophages, myeloid-derived suppressor cells, and regulatory T cells. Despite the presence of these T cells, the ascitic microenvironment exerts profound functional suppression through multiple converging mechanisms: soluble inhibitory factors (e.g., TGF-β, IL-10, VEGF), metabolic constraints (e.g., glucose deprivation, lactate accumulation), and cell-contact-dependent checkpoints (e.g., PD-1/PD-L1, CTLA-4), hence it promotes local immunosuppression and tumor persistence (10, 11). Therefore, the immunosuppressive peritoneal microenvironment, which favors innate immunity while actively suppressing adaptive immunity and enabling tumor cells to evade antigen-specific host immune surveillance likely represents one important factor underlying the limited efficacy of systemic immunotherapy against peritoneal metastases. Additional resistance mechanisms, including physical barriers to drug penetration, tumor heterogeneity, and systemic immune dysfunction, may also play substantial roles. Single-cell profiling of ovarian cancer has substantiated this view by demonstrating that ascites-enriched CD8^+^GZMK^+^ memory T cells share T cell receptors with tumor-infiltrating exhausted CD8^+^T cells in both primary and metastatic lesions, suggesting that the ascitic T-cell pool may serve as a reservoir for tumor-infiltrating lymphocytes. The same study further revealed that tumor-associated macrophages in peritoneal metastases originate predominantly from circulating monocytes, whereas ascites-resident macrophages are largely of embryonic origin, illustrating the ontogenetic heterogeneity of myeloid cells across peritoneal compartments (12). Consequently, therapeutic paradigms are shifting from palliative symptom control toward integrated, synergistic approaches that simultaneously reprogram the immunosuppressive ascitic niche and directly eradicate tumor cells. Such strategies represent a potential direction to overcome current clinical bottlenecks and achieve better survival outcomes.

This narrative review provides a focused summary of recent progress in the study and management of malignant ascites, emphasizing the relationships among immune subsets, soluble mediators, and stromal components in the ascitic immune microenvironment. The literature covered was selected based on clinical relevance, mechanistic novelty, and representation of major therapeutic modalities. From this literature, we derive a conceptual framework for the rational design of next-generation immunotherapies for peritoneal malignancies.

## 2 *In situ* vaccination strategies for malignant ascites

Activating endogenous antigen presentation to initiate tumor-specific T-cell responses is a powerful and well-defined strategy for remodeling the immunosuppressive ascitic microenvironment, and the prototypical example of this is the “*in situ* vaccination” effect (a strategy that induces tumor-specific immune responses at the tumor site) induced by oncolytic viruses (OVs), which selectively infect and lyse tumor cells while also inducing immunogenic cell death (ICD), thereby promoting the efficient release of tumor-associated antigens (TAAs). Damage-associated molecular patterns (DAMPs) and inflammatory cytokines initiate a cascade that promotes dendritic cells (DCs) recruitment, antigen uptake, processing, and cross-presentation, thus converting the immunologically “cold” ascitic niche into an inflamed, T-cell-infiltrated “hot” microenvironment. As the most potent professional antigen-presenting cells, DCs serve as central orchestrators of anti-tumor immunity: they prime naïve T cells, drive clonal expansion of tumor-specific CD8^+^ T cells, and are indispensable mediators in reshaping the ascitic immune landscape.

This section covers two distinct but mechanistically convergent strategies that achieve *in situ* immune activation in the ascitic microenvironment: oncolytic viruses, which induce immunogenic tumor cell lysis and endogenous antigen release at the tumor site, and dendritic cell vaccines, which deliver ex vivo-loaded tumor antigens to lymph nodes to prime T-cell responses. Although these approaches differ in their composition and mode of delivery, both aim to generate tumor-specific T-cell immunity through endogenous antigen presentation.

### 2.1 Intraperitoneal administration of oncolytic viruses

The evidence base for intraperitoneal oncolytic virotherapy spans from preclinical models to early-phase clinical trials, with no agent yet approved specifically for malignant ascites. The clinical studies discussed below are single-arm phase II trials, and no randomized controlled data are available for oncolytic viruses in this setting. With respect to clinical efficacy, a phase II trial in patients with malignant ascites has shown that intraperitoneal administration of the oncolytic virus H101 prolongs median time to repeat paracentesis (TTRP), defined as the interval from the baseline paracentesis to the first post-treatment therapeutic paracentesis or death (**Table 1**). This trial specifically enrolled patients with malignant ascites secondary to pancreatic, gastrointestinal, ovarian, or biliary cancer. Moreover, single-cell RNA sequencing analysis revealed that the ascitic tumor microenvironment (TME) of treatment-responsive patients was highly conducive to anti-tumor immune activation.H101 induced potent oncolysis and early-phase viral replication, and thereby caused a substantial proliferative expansion of CXCR6 ^+^ GZMK^+^ CD8 ^+^ T cells, which are naturally enriched for tumor reactivity and have enhanced cytotoxic function. H101 also reprogrammed myeloid populations toward an immunostimulatory phenotype and promoted immunological checkpoint-mediated crosstalk between CD8^+^ T cells and antigen-presenting myeloid cells, providing a mechanistic rationale for combination therapy with immune checkpoint inhibitors(ICIs) (13). A subsequent meta-analysis, which included predominantly single-arm studies, reported an overall response rate of 69.9% for malignant serous cavity effusions treated with either H101 monotherapy or combination regimens, with a safety profile that appears favorable and manageable (14).

**Table 1:** Selected clinical studies of intraperitoneal or local immunotherapies for malignant ascites.

(A) Completed studies with published results					
Study identifier	Tumor Type	Enrolled population	Drug category	Drug name	Mechanism
NCT00836654 (28)	EpCAM^+^ carcinomas (gastric, ovarian, colorectal)	Malignant ascites due to EpCAM^+^ epithelial cancer	T-cell engager	Catumaxomab	Trifunctional EpCAM×CD3 antibody
NCT00822809 (CASIMAS) (56)	EpCAM^+^ carcinomas	Malignant ascites due to epithelial cancer	T-cell engager	Catumaxomab (+ prednisolone)	Trifunctional EpCAM×CD3 antibody
NCT06266091 (32)	Gastric, colorectal, ovarian	Malignant ascites due to epithelial cancer	T-cell engager	M701	Anti-EpCAM × anti-CD3 bispecific TCE
NCT05745233 (19)	Pancreatic (60%), GI, ovarian, biliary	Malignant ascites due to gastrointestinal or pancreaticobiliary tract cancer	Immune checkpoint blockade	Nivolumab	Anti-PD-1 mAb
ChiCTR2100043683 (20)	HCC, CCA	Malignant ascites due to primary liver cancer	Immune checkpoint blockade	Sintilimab	Anti-PD-1 mAb
NCT04771676 (13)	Gastric, pancreatic, biliary	Malignant ascites due to pancreatic, gastrointestinal, ovarian, or biliary cancer	Oncolytic adenovirus	H101	Selective oncolysis + ICD + T-cell activation
NCT01852409 (48)	Advanced solid tumors with ascites	Refractory malignant ascites due to advanced solid tumors	Anti-angiogenic	Bevacizumab	Anti-VEGF-A mAb
(B) Ongoing or recently completed trials (published results available)					
NCT06432296	Advanced epithelial solid tumors	Malignant ascites due to advanced epithelial solid tumors	T-cell engager	M701	Anti-EpCAM × anti-CD3 bispecific TCE
NCT05501340 (21)	Gastric (46%), CRC, mesothelioma	Cancerous ascites due to advanced refractory solid tumors	Immune checkpoint blockade + PRaG	Serplulimab (HLX10)	Anti-PD-1 mAb + radiotherapy + GM-CSF
(A) Completed studies with published results					
Study identifier	Phase/Design	Sample size	Intervention		Route
NCT00836654 (28)	Phase II/III; randomized, open-label	N=258 (170 C+P; 88 P alone)	Four escalating IP infusions (10, 20, 50, 150 µg) on Days 0, 3, 7, 10		IP
NCT00822809 (CASIMAS) (56)	Phase IIIb; randomized, open-label	N=219	3-h IP infusion ± prednisolone 25mg premedication		IP
NCT06266091 (32)	Phase II; randomized, controlled, open-label	N=84 (43 M701; 41 control)	50-400 µg IP on Days 1,4,11,18; optional Q2W maintenance		IP
NCT05745233 (19)	Phase I/II; single-arm, open-label, single-center	N=9	20 mg IP after each therapeutic paracentesis		IP
ChiCTR2100043683 (20)	Phase Ib; single-arm, open-label, single-center	N=21	100 mg IP weekly		IP
NCT04771676 (13)	Phase II; single-arm, open-label, single-center	N=25	1.5×10^12^ vp IP on Day 1 and Day 3		IP
NCT01852409 (48)	Phase I; single-arm, open-label, single-center	N=13	2.5-7.5 mg/kg IP weekly		IP
(B) Ongoing or recently completed trials (published results available)					
NCT06432296	Phase III; randomized, controlled, open-label, multicenter	Planned (ongoing)	IP M701 infusion vs. paracentesis alone		IP
NCT05501340 (21)	Phase I/II; single-arm, open-label, multicenter	Planned (dose escalation)	PRaG regimen + IP serplulimab		IP
(A) Completed studies with published results					
Study identifier	Primary endpoint (definition)	Key efficacy outcome	Grade ≥3 AEs		Key limitations
NCT00836654 (28)	PuFS: time from treatment initiation to first need for therapeutic paracentesis or death, whichever occurred first	Median PuFS: 46 vs. 11 days (HR = 0.254; *p* < 0.0001); mOS: 72 vs. 68 days (*p* = 0.0846)	~80% any-grade; Grade ≥3 manageable		Drug withdrawn from EU market (2017) for commercial reasons; re-approved in 2025
NCT00822809 (CASIMAS) (56)	Composite safety score: sum of worst-grade scores (CTCAE v3.0) for pyrexia, nausea, vomiting, and abdominal pain between first infusion and 28 days after last infusion	Median PuFS: 37 days (no prednisolone) vs. 30 days (+ prednisolone); mOS: 86 vs. 124 days	Comparable between arms; ~48% Grade ≥3		Prednisolone did not reduce AEs; open-label design
NCT06266091 (32)	PuFS: time from randomization to first re-puncture due to ascites intolerance or death, whichever occurred first	Median PuFS: 75 vs. 25 days (*p* = 0.0065, HR = 0.43); mOS: 110 vs. 76 days (HR = 0.68, *p* = 0.1443)	52% (M701) vs. 55% (control); 2 CRS cases (both grade 2)		Small sample size; RLC≥13% subgroup had greater benefit; QoL data not collected
NCT05745233 (19)	Ascites response rate: reduction in ascitic fluid volume with prolonged paracentesis intervals (clinical response per treating physician assessment)	Clinical response rate 77.8%; reduced ascites volume; prolonged paracentesis interval	None grade ≥3		Very small sample size (n=9); single-arm; single-center; short follow-up
ChiCTR2100043683 (20)	Objective ascites response rate: CR (complete disappearance of ascites for ≥4 weeks) or PR (>50% reduction in ascites depth by ultrasound for ≥4 weeks) per WHO criteria	Objective response rate 43.75%; mOS 17.6 weeks	33.3% (fatigue, rash, abdominal pain); no grade ≥4		Single-arm; single-center; heavily pretreated cohort (57.1% ECOG 3; 47.6% Child-Pugh C)
NCT04771676 (13)	TTRP (time to repeat paracentesis): interval from baseline paracentesis to first post-treatment therapeutic paracentesis or death, whichever occurred first	Median TTRP: 45 days (95% CI 16.5-73.5); mOS: 58 days	8% (abdominal pain, anemia)		Single-arm; single-center; small sample size; heterogeneous tumor types
NCT01852409 (48)	MTD determination: incidence of dose-limiting toxicity (DLT) per standard 3 + 3 dose-escalation design	No MTD identified; DCR 61.5%; mOS 103 days	7.7% (grade 3 bowel obstruction; n=1)		Phase I dose-escalation only (n=13); no efficacy endpoint; single-arm
(B) Ongoing or recently completed trials (published results available)					
NCT06432296	PuFS: time from randomization to first re-puncture or death, whichever occurred first	Phase II results: median PuFS 75 vs. 25 days (*p* = 0.0065); mOS 110 vs. 76 days (HR = 0.68)	No additional SAEs; anemia and low WBC manageable		Pivotal phase III ongoing; NDA submitted in China (2026)
NCT05501340 (21)	Safety and efficacy: phase I—incidence of DLT; phase II—overall survival (OS)	Dose-escalation ongoing	Not yet reported		Ongoing; dose-escalation phase

C+P, catumaxomab plus paracentesis; P, paracentesis alone; HCC, hepatocellular carcinoma; CCA, cholangiocarcinoma; GI, gastrointestinal; CRC, colorectal cancer; IP, intraperitoneal; PuFS, puncture-free survival; TTRP, time to repeat paracentesis; mOS, median overall survival; OS, overall survival; DCR, disease control rate; MTD, maximum tolerated dose; DLT, dose-limiting toxicity; PRaG, radiotherapy combined with GM-CSF; RLC, relative lymphocyte count; QoL, quality of life; SAE, serious adverse event; NDA, new drug application; CTCAE, Common terminology criteria for adverse events; CRS, cytokine release syndrome; CR, complete response; PR, partial response; NA, not available.

Beyond directly lysing tumor cells to induce ICD and release TAAs, OVs can also reshape the cellular composition and functional polarization of immune cells within malignant ascites. This has been nicely demonstrated in the ID8 murine ovarian cancer model, where intraperitoneal administration of an oncolytic adenovirus greatly enhanced CD8 ^+^ T-cell infiltration into ascites. The authors showed that the virus promoted proliferation and differentiation of cells toward an effector phenotype, thereby increasing IFN-γ production, while at the same time inducing repolarization of M2-like tumor-associated macrophages (TAMs) toward an M1-like pro-inflammatory state, as demonstrated by upregulation of MHC class II and reduced secretion of immunosuppressive mediators such as IL-6 (8). Moreover, OVs can drive systemic immune reprogramming via modulation of tumor-derived extracellular vesicles (EVs). Specifically, infection of ovarian cancer cells with an oncolytic rhabdovirus greatly increased the release of EVs enriched in immunomodulatory microRNAs (e.g., miR-19a), which upon uptake by TAMs reversed TAM-mediated immunosuppression, enhanced CD8 ^+^ T-cell anti-tumor activity, and ultimately led to a significant prolongation of survival in tumor-bearing mice (15).

Since the studies reviewed earlier have established that genetic engineering of OVs to express immunomodulatory molecules is a very promising approach for enhancing anti-tumor immunity, the work of Freedman et al. (16) is discussed below, who developed an oncolytic adenovirus expressing a bispecific T-cell engager (BiTE) directed against fibroblast activation protein (FAP), named EnAd-FAP-BiTE, and rigorously tested its immunomodulatory activity in a preclinical model of malignant ascites. Since the virus mediates localized production of FAP-BiTE, which recruits and reactivates endogenous T cells, thereby selectively eliminating FAP-high cancer-associated fibroblasts (CAFs), EnAd-FAP-BiTE exerts dual anti-tumor mechanisms: direct oncolysis and stromal-immune remodeling. In patient-derived malignant ascites samples, EnAd-FAP-BiTE treatment reversed T-cell exhaustion, as evidenced by reactivation of PD-1^+^ T cells, and significantly reduced the frequency of FAP^+^ CAFs. This was accompanied by decreased TGF-β levels and upregulated expression of pro-inflammatory cytokines, including IFN-γ and IL-2. Moreover, TAMs underwent phenotypic repolarization from the immunosuppressive M2 state (CD163^+^CD206^+^) toward the immunostimulatory M1 phenotype (increased CD86 expression), collectively indicating a systemic shift of the ascitic microenvironment from immunosuppression to immune activation. For this reason, these findings indicate that OVs are not simply cytolytic agents but bona fide *in situ* multifunctional immunotherapeutic platforms.

### 2.2 DC vaccines activate anti-tumor T cells

Clinical evidence for DC vaccines in malignant ascites is currently limited to case reports, and no prospective or randomized data are available. Ultrasound-guided intranodal injection of an individualized neoantigen peptide-loaded DC vaccine into the inguinal lymph nodes of a patient with chemotherapy-refractory ovarian cancer and malignant ascites resulted in a reduction of tumor cells in the ascitic fluid, a drop in serum CA-125 levels, and clinical benefit with regard to dyspnea and abdominal discomfort. Immunomonitoring established that the vaccine induced antigen-specific CD8^+^ T-cell responses against the PPM1F mutant neoantigen in peripheral blood, and T-cell receptor (TCR) β-chain sequencing showed clonal expansion of neoantigen-reactive T-cell clones with preferential infiltration into the ascites-derived TME, thereby providing direct evidence of vaccine-driven localized immune remodeling (17).

In a patient with end-stage duodenal adenocarcinoma complicated by peritoneal carcinomatosis and malignant ascites, combination immunotherapy using DC vaccines pulsed with WT1 and MUC1 peptides and adoptively transferred CD3-activated T lymphocytes led to ascites resolution, improvement in performance status (ECOG score changing from 3 to 0), no treatment-related adverse events, and remained recurrence-free for 54 months. This case demonstrates that even in rare, chemotherapy-refractory malignancies, DC vaccine-driven activation of T cells against broadly expressed TAAs can restore anti-tumor immune surveillance within the immunosuppressive ascitic microenvironment (18). Nevertheless, these observations are derived from single case reports and await validation in larger patient cohorts.

## 3 Intraperitoneal administration of immune checkpoint blockade for malignant ascites

The peritoneal cavity is far from being just a passive anatomical compartment for peritoneal metastasis; it is actually a functionally active lymphoid organ with well-defined immunological properties, containing a rich, organized population of immune cells including macrophages, T cells, B cells, and DCs, which form specialized structures called “milky spots” serving as major antigen-processing hubs in the peritoneum. However, during malignant ascites formation, tumor cells exploit the programmed cell death protein 1 (PD-1)/programmed death ligand 1 (PD-L1) immune checkpoint pathway to evade host immunity: by upregulating PD-L1, they engage PD-1 on tumor-infiltrating T cells, inducing T-cell exhaustion and functional impairment. Thus, the PD-1/PD-L1 axis is established as a fundamental immune suppression mechanism in the peritoneal microenvironment and a highly attractive target for localized immunotherapeutic intervention. Even more importantly, intraperitoneal drug delivery allows for sustained, high-concentration therapeutic exposure in the peritoneal cavity while avoiding the systemic immune-related adverse events common with conventional intravenous immunotherapy (19).

### 3.1 Targeting the PD-1/PD-L1 axis to reverse immunosuppression in malignant ascites

The intraperitoneal administration of PD-1 inhibitors, namely sintilimab in hepatocellular carcinoma (HCC) patients with malignant ascites, has been evaluated in a single-arm, single-center phase Ib trial, which enrolled a clearly defined, heavily pretreated, advanced-disease cohort: 57.1% of the patients had an ECOG performance status of 3, and 47.6% were Child-Pugh C, indicating severe hepatic dysfunction. Although intraperitoneal sintilimab has a high-risk profile, it showed favorable safety data, namely no treatment-related grade ≥ 4 adverse events, and achieved an objective ascites response rate of 43.75% (20). This trial specifically enrolled patients with malignant ascites due to primary liver cancer. Wang et al. (19) subsequently conducted a single-arm, early-phase prospective study on intraperitoneal nivolumab (20 mg after each therapeutic paracentesis) in patients with malignant ascites secondary. This study specifically enrolled patients with malignant ascites secondary to gastrointestinal or pancreaticobiliary tract cancer. The regimen described for gastrointestinal or pancreaticobiliary malignancies produced a clinical response rate of 77.8%, with clear evidence of reduced ascites volume and prolonged time intervals between paracenteses. The only adverse events observed were mild and transient grade 1 puncture-site tenderness, and no grade ≥ 3 toxicities were noted; therefore, the study provides preliminary support for the safety, feasibility, and palliative benefit of low-dose intraperitoneal PD-1 blockade. Both studies are limited by their single-arm design and small sample sizes (21 and 9 patients, respectively), and the observed response rates require confirmation in larger prospective trials.

Additionally, various immunosuppressive cell populations—regulatory T cells (Tregs), TAMs, and myeloid-derived suppressor cells (MDSCs)—coexist within the ascitic microenvironment and collectively establish a localized immunosuppressive network. Emerging evidence indicates that PD-1 is not only highly expressed on exhausted T cells but also upregulated on TAMs. PD-1^+^ TAMs predominantly adopt an M2-like, pro-tumorigenic phenotype; blockade of the PD-1/PD-L1 axis restores their phagocytic capacity and reprograms them toward an anti-tumor functional state, enhancing local immune surveillance. An ongoing multicenter phase II trial (PRaG 4.0P) is evaluating a combinatorial intraperitoneal strategy that integrates immune checkpoint blockade with an *in situ* vaccination effect—designed to remodel the ascitic immune landscape and improve ascites control and long-term survival outcomes. Preliminary dose-escalation data from this trial will inform optimization of dosing regimens and provide pivotal evidence for standardizing intraperitoneal immune checkpoint blockade (ICB) therapy (21). Beyond the PD-1/PD-L1 axis, specific T-cell subsets in ascites may inform treatment stratification. In ovarian cancer, ascites-resident mucosal-associated invariant T (MAIT) cells, along with IL13RA1^+^ and VCAM1^+^ endothelial subsets in primary tumors, have been identified as potential predictors of platinum-based chemotherapy response. Whether these MAIT cells also predict response to intraperitoneal checkpoint blockade warrants further investigation (12). Prior clinical experience with intraperitoneal drug delivery underscores that dose selection must carefully balance therapeutic efficacy with local tolerability and systemic safety. **Table 1** summarizes key completed and ongoing clinical trials of intraperitoneal immunotherapy for malignant ascites, including the three ICB-based studies highlighted above.

### 3.2 Investigating multi-mechanism synergistic ICB therapies to overcome primary resistance

Building upon the clinically validated safety and preliminary efficacy of intraperitoneal immunotherapy, preclinical research is actively exploring rational combination strategies to overcome primary resistance to ICB therapy. The following strategies remain at the preclinical stage and have not yet progressed to clinical evaluation in patients with malignant ascites.

Chicoric acid (CA), a natural phenolic compound with immunostimulatory activity, promotes lymphocyte proliferation and enhances CD8^+^ T-cell infiltration into the TME. In a patient-derived xenograft (PDX) model of ovarian cancer, CA synergized with anti-PD-1/PD-L1 antibodies to significantly augment anti-tumor efficacy (22). Celastrol—formulated in an injectable oil-in-water emulsion hydrogel (Gel@Cel)—induces ICD in tumor cells, activating DCs and promoting effector T-cell infiltration in a low-immunogenic HCC ascites model. Although celastrol triggers compensatory upregulation of PD-L1 on tumor cells, its combination with anti-PD-1 antibodies not only counteracts this adaptive immune resistance but also amplifies anti-tumor immunity, effectively converting immunologically “cold” tumors into “hot” tumors (23).

The chimeric orthopoxvirus CF33-hNIS-antiPDL1 not only efficiently infects and lyses ex vivo ascites-derived cancer cells from gastric cancer patients with peritoneal metastasis but also—via its encoded anti-PD-L1 single-chain variable fragment (scFv)—blocks PD-L1 on the surface of tumor cells. Concurrently, it reduces key immunosuppressive factors—EGF, PDGF, IL-10—in the ascitic microenvironment and upregulates the cytotoxic activation marker CD107α on CD8^+^ T cells, demonstrating dual oncolytic and immunomodulatory activity (24). Moreover, ferroptosis-inducing strategies potentiate the efficacy of ICB. An injectable hydrogel covalently conjugated with sulfasalazine (CH-OD-SSZ) triggers immunogenic ferroptosis in tumor cells. In a murine hepatocellular carcinoma-associated malignant ascites model, combination therapy with CH-OD-SSZ and anti-PD-1 antibodies achieved complete ascites resolution in over 50% of mice and established durable, antigen-specific immune memory (25).

## 4 T-cell engagers and adoptive cellular therapies for malignant ascites

TCEs are bispecific antibodies that simultaneously bind a tumor-associated antigen—e.g., epithelial cell adhesion molecule (EpCAM)—and the CD3 component of the TCR complex. By bridging cytotoxic T lymphocytes and tumor cells, TCEs induce potent, MHC-unrestricted T-cell activation and targeted tumor cell lysis. This mechanism holds particular promise for treating malignant ascites, a challenging complication of advanced abdominal malignancies characterized by immunosuppressive, T-cell-excluded microenvironments.

Based on this mechanism, the clinical feasibility of TCEs has been validated in multiple studies, catumaxomab, the EpCAM×CD3-targeting trifunctional antibody, is the most representative example. Catumaxomab has two clearly defined antigen-binding arms: one that binds EpCAM on tumor cells and the other that binds CD3 on T cells, and it possesses a unique chimeric Fc region made up of murine IgG2a and rat IgG2b heavy chains which allows it to bind with high affinity to human Fcγ receptors I and III (FcγRI/III) on accessory immune cells (e.g., macrophages, DCs, NK cells). Thus, it enables a well-orchestrated immune response (26). The tumor site exhibits a multimodal immune response that is captured by the “trifunctional” architecture of this molecule, which confers cytotoxic potency more than 1,000-fold greater than that of conventional EpCAM-targeting monoclonal antibodies (27).

### 4.1 Clinical application of catumaxomab

As the first T-cell-engaging bispecific antibody to be authorized for malignant ascites, catumaxomab demonstrated clinically meaningful efficacy in its pivotal phase II/III trial. Intraperitoneal administration of catumaxomab at four escalating doses (10, 20, 50, and 150 µg) together with large-volume paracentesis significantly prolonged puncture-free survival (PuFS) compared with paracentesis alone (median, 46 vs. 11 days; HR = 0.254; *p* < 0.0001) and extended the median time to next therapeutic paracentesis from 13 to 77 days (HR = 0.169; *p* < 0.0001) (28). In the prespecified subgroup of patients with platinum-resistant or chemotherapy-refractory ovarian cancer, catumaxomab conferred a clinically meaningful benefit: median PuFS was 29.5 days, and 22% of these patients achieved at least a fourfold prolongation in the interval between consecutive paracenteses (29). The CASIMAS trial specifically enrolled patients with malignant ascites due to epithelial cancer. Regarding overall survival (OS), the pivotal trial demonstrated a positive trend in the overall population (median 72 vs. 68 days; HR = 0.723; *p* = 0.0846), with a statistically significant OS benefit observed specifically in the gastric cancer subgroup (median 71 vs. 44 days; HR = 0.469; *p* = 0.0313) (28). Catumaxomab received initial European marketing authorization in April 2009; however, in June 2017, the authorization was withdrawn at the request of the marketing authorization holder for commercial reasons, as the product had not been actively marketed in the EU since 2014. The rights were subsequently acquired by Lindis Biotech, and catumaxomab was reauthorized by the European Commission in February 2025 for the intraperitoneal treatment of malignant ascites in adults with EpCAM-positive carcinomas who are not eligible for further systemic anticancer therapy.

In addition to direct tumor cell lysis, catumaxomab has well-characterized immunomodulatory properties. Intraperitoneal catumaxomab induces sustained peripheral T-cell activation, as documented by upregulation of CD69 and HLA-DR (30). Th1-polarized CD4^+^ and CD8^+^ T cells expressing CXCR3 transiently decrease in peripheral blood—likely reflecting recruitment to the peritoneal cavity—and return to baseline four weeks after treatment (30). This is supported by the observation that EpCAM-specific T cells are dramatically enriched in ascitic fluid of patients with postoperative non-malignant ascites, providing functional evidence of antigen-driven T-cell homing to the peritoneal compartment (30).

Catumaxomab also induces durable immune memory and epitope spreading (30, 31). EpCAM-specific T cells, present at low frequencies and in an exhausted state before treatment, display a biphasic kinetic pattern following intraperitoneal administration, with re-expansion peaking approximately four weeks after treatment cessation (30).

Treatment of certain patients also results in *de novo* antibody responses against cancer-testis antigens, indicating that catumaxomab-mediated tumor cell lysis primes adaptive immunity against a broader repertoire of tumor antigens (31). In the catumaxomab pivotal trial, the primary efficacy endpoint was puncture-free survival (PuFS), defined as the time from treatment initiation to the first need for therapeutic paracentesis or death, whichever occurred first. The secondary endpoint time to next puncture (TTPu) measured the interval from catheter removal to the first subsequent therapeutic puncture, without censoring for death as a competing event. In subsequent catumaxomab studies, PuFS and TTPu were similarly defined, providing consistency across the pivotal and CASIMAS phase IIIb trials.

### 4.2 Clinical breakthroughs with novel TCEs

A phase II trial of M701, a newly developed bispecific TCE antibody, has demonstrated the therapeutic potential of the approach. In this open-label, multicenter, randomized, controlled phase II trial (NCT06266091), 84 patients with moderate- to large-volume malignant ascites due to gastric, colorectal, or ovarian cancer were randomly assigned at a 1:1 ratio to receive paracentesis plus intraperitoneal M701 infusion (n=43) or paracentesis alone (n=41); both arms received systemic anticancer therapy according to Chinese clinical guidelines. The primary endpoint was puncture-free survival (PuFS), defined as the time to first re-puncture or drainage due to intolerance of malignant ascites or death after the last drainage from days 1 to 18, whichever occurred first. Median PuFS was 75 days in the M701 group versus 25 days in the control group (HR = 0.43; 95% CI: 0.22-0.81; *p* = 0.0065). A trend toward improved overall survival was observed (median 110 vs. 76 days; HR = 0.68; *p* = 0.1443). In a prespecified subgroup analysis, patients with baseline peripheral blood relative lymphocyte count ≥13% derived the greatest benefit, with a median PuFS of 125 days and significantly prolonged overall survival (152 vs. 76 days; *p* = 0.0155) (32). A phase III randomized controlled trial (NCT06432296) is currently ongoing to confirm these findings.

Besides EpCAM, TRAIL-R2 (also designated DR5), the receptor for tumor necrosis factor-related apoptosis-inducing ligand (TRAIL), has become an extremely attractive novel target for TCE therapies because it is preferentially overexpressed on a wide variety of tumor cells, such as those from ovarian, gastric, and colorectal carcinomas, while being expressed at very low levels on most normal tissues. Satta et al. (33) developed a bispecific single-chain diabody directed against TRAIL-R2×CD3 and rigorously demonstrated its functional activity in a primary ex vivo model using ascites-derived cells from ovarian cancer patients, showing clearly that the construct potently activated ascites-resident CD8^+^ T cells as evidenced by upregulation of activation and degranulation markers CD107a and CD137, while simultaneously inducing selective apoptosis in EpCAM^+^ tumor cells. Thus, this paper is the first to report TRAIL-R2 incorporation into the TCE platform, and it opens new therapeutic avenues for malignant ascites arising from EpCAM-negative or EpCAM-low tumors.

### 4.3 Reporting and discussion of TCE-related adverse events

Adverse events related to intraperitoneal TCEs are for the most part mild to moderate and easily managed, with fever, nausea, vomiting, and abdominal pain being the most commonly reported events, which are all consistent with localized immune activation resulting from T-cell engagement in the peritoneal cavity. In the pivotal catumaxomab trial, cytokine release syndrome (CRS) occurred in approximately 72% of treated patients; the majority of cases were grade 1–2 and reversible, although life-threatening systemic inflammatory response syndrome (SIRS) was reported in 0.6% of patients. The occurrence of CRS has been positively correlated with clinical efficacy in some studies (28).

As a trifunctional, rat/mouse hybrid monoclonal antibody (not a conventional chimeric antibody), the immunogenicity-induced human anti-mouse antibody (HAMA) response was long considered a major limitation to clinical use of catumaxomab. But a *post hoc* analysis of the pivotal phase II/III trial showed that patients who developed HAMA by Day 8 after treatment had significantly longer median paracentesis-free survival. HAMA-positive patients had longer time to next paracentesis and better overall survival than HAMA-negative patients. In the ovarian cancer subgroup, the median overall survival was 163 days in HAMA-positive patients compared to 82 days in HAMA-negative patients (*p* = 0.0123). More importantly, HAMA-positive patients had significantly higher treatment compliance (98% vs. 85%, *p* = 0.0292), which strongly suggests that the full therapeutic regimen was better tolerated by HAMA-positive patients. Therefore, these data suggest that HAMA formation is not simply a neutralizing immune response but rather reflects robust activation of the host’s functional immune system, making it a clinically relevant surrogate biomarker for favorable outcomes (31).

Because intraperitoneal delivery does not introduce new or greater safety risks than systemic administration and its overall safety profile is well characterized, predictable, and easily monitored with effective supportive measures in place, intraperitoneal TCE therapy offers potent local tumor clearance in malignant ascites without compromising the favorable benefit-risk balance. The safety profile of M701 differs from that of catumaxomab. In the randomized phase II trial, CRS occurred in 4% of M701-treated patients, all grade 1-2, and no grade ≥3 CRS was reported (32). SAE incidence was comparable between the M701 and control groups, and no patient discontinued M701 due to severe adverse reactions (32). In contrast, catumaxomab was associated with CRS in approximately 72% of patients, life-threatening SIRS in 0.6%, and HAMA formation in most patients (28, 31). These differences should be considered when selecting T-cell engager therapy. Thus, it represents a highly promising and clinically viable immunotherapeutic strategy (29, 32).

### 4.4 Adoptive cellular therapies for malignant ascites

Adoptive transfer of NK cells and activated T cells in malignant ascites has been briefly mentioned in the Introduction and Section 1.2. This subsection provides a focused discussion of CAR-T, CAR-NK, TIL/TAL, and γδ T cell approaches. Adoptive cellular therapy (ACT) is a distinct immunotherapeutic approach in which immune effector cells are expanded or genetically engineered ex vivo and subsequently reinfused into patients. Unlike T-cell engagers, which redirect endogenous T cells, ACT delivers a defined cellular product with potential antigen-specific or innate anti-tumor activity. The peritoneal cavity is an accessible compartment for locoregional delivery of cellular therapies, offering the potential for high local effector-to-target ratios while limiting systemic exposure. The evidence for ACT in malignant ascites remains at an early stage and spans case reports, small early-phase trials, and preclinical studies.

At the case-report level, the most direct clinical evidence for CAR-T cells in malignant ascites comes from a case report of a patient with relapsed follicular lymphoma and massive chylous ascites who received CD19-directed CAR-T cells generated using the Piggy Bac transposon system (34). The patient achieved a nearly complete metabolic remission at day 28, with grade 1 cytokine release syndrome and no neurotoxicity. CAR-T cells were detected in the ascites at higher absolute numbers than in peripheral blood on day 7 after infusion and remained detectable in ascites on day 21, indicating trafficking of CAR-T cells into the peritoneal compartment and local expansion. This observation is limited by its single-case nature and by the distinct biology of chylous ascites compared with malignant ascites arising from solid tumors.

At the preclinical level, evidence supports the rationale for intraperitoneal CAR-T cell delivery in peritoneal carcinomatosis. In mouse models of gastroesophageal cancer peritoneal carcinomatosis that recapitulated ascites, bowel obstruction, and an immunosuppressive microenvironment with elevated ascitic TGF-β and tumor-cell PD-L1 expression, intraperitoneal administration of a mesothelin-targeted CAR-T construct incorporating a PD-1 dominant-negative receptor achieved superior anti-tumor efficacy and longer survival compared with intravenous administration, even at a fivefold lower dose (35). Intraperitoneally administered CAR-T cells also exhibited functional persistence upon tumor rechallenge and were effective against distant disease sites. These findings provided the rationale for an ongoing phase I trial (NCT06623396) evaluating intraperitoneal mesothelin-targeted CAR-T cells in patients with gastroesophageal cancer peritoneal carcinomatosis. These data are preclinical, and clinical safety and efficacy data are not yet available.

At the early-phase clinical level, evidence for CAR-NK cells in malignant ascites is limited to a phase I pilot study (NCT03415100) that evaluated NKG2D ligand-targeted CAR-NK cells in three patients with chemotherapy-refractory metastatic colorectal cancer (36). CAR-NK cells were generated by mRNA electroporation, conferring transient CAR expression, and were administered intraperitoneally in two patients with malignant ascites. Reduction in ascites volume and a decrease in EpCAM-positive tumor cells in ascites were observed after low-dose intraperitoneal infusion. The third patient, who had liver metastases without ascites, received ultrasound-guided percutaneous injection of CAR-NK cells into liver lesions, with a complete metabolic response observed at the injection site. No dose-limiting toxicities or high-grade cytokine release syndrome occurred. This study is limited by its small sample size (n=3), single-arm design, and short follow-up. The use of mRNA electroporation for transient CAR expression may offer a safety advantage but necessitates repeated infusions.

At the non-randomized prospective level, TIL and TAL approaches have the largest clinical dataset among ACT modalities in malignant ascites. A prospective study enrolled 32 patients with ovarian cancer and malignant ascites, with 8 patients receiving TALs alone, 13 receiving chemotherapy alone, and 11 receiving combined TALs and chemotherapy (37). The objective ascites response rate was 90.9% in the combination group, compared with 37.5% in the TALs-alone group and 53.8% in the chemotherapy-alone group. Median time to progression was 13 months in the combination group, compared with 1 month in the TALs-alone group (*p* < 0.001) and 6 months in the chemotherapy-alone group (*p* = 0.027). Median overall survival was 25 months in the combination group versus 7 months in the TALs-alone group (*p* < 0.001), but the difference versus chemotherapy alone (18 months) was not statistically significant (*p* = 0.135). No treatment-related toxicities were attributed to intraperitoneal TALs administration. This study is limited by its non-randomized design, small sample size, and single-center conduct.

A methodology study established a protocol for expanding TILs from malignant pleural effusion and ascites using engineered cells for costimulatory enhancement (ECCE), a K562-derived feeder cell line expressing 4-1BBL, in combination with IL-2, IL-7, and IL-15 (38). This approach yielded TIL expansion with a less differentiated effector memory phenotype and enhanced IFN-γ and TNF-α secretion compared with IL-2 alone. These findings are preclinical and require validation in clinical trials.

At the *in vitro* level, Vδ2+ γδ T cells are innate-like lymphocytes with MHC-unrestricted anti-tumor activity. An *in vitro* study isolated Vδ2+ T cells from ascites of patients with ovarian cancer, fallopian tube cancer, or primary peritoneal cancer and expanded them with zoledronate and IL-2 (39). Vδ2+ T cells expanded from ascites exhibited cytotoxicity against autologous tumor cells, with disappearance of mixed tumor cells in a subset of cases. The proliferation rate of Vδ2+ T cells from ascites was lower than that from peripheral blood, and expansion efficiency varied among patients. These findings are limited to *in vitro* studies, and clinical data are lacking.

Collectively, ACT approaches for malignant ascites remain at an early stage of development. CAR-T and CAR-NK cell therapies are supported by case-report-level or small phase I evidence, while TIL/TAL approaches have the largest clinical dataset but lack randomized validation. The intraperitoneal route is consistently used across these studies, leveraging the accessibility of the peritoneal cavity and the potential for localized immune activation. Future studies will need to address the immunosuppressive ascitic microenvironment, optimize cell manufacturing and dosing, and establish standardized endpoints for clinical evaluation.

## 5 Targeting TAMs and other immune cell-related pathways for malignant ascites

In peritoneal carcinomatosis, the ascitic microenvironment exhibits a distinct immunosuppressive landscape characterized by profound adaptive immunity impairment. Central to this immunosuppression are TAMs, MDSCs and the dysregulated cytokine network they orchestrate. Specifically, TAMs in this setting are almost universally polarized toward an M2-like, pro-tumorigenic phenotype, whereas MDSCs directly inhibit T-cell activation and effector function by the secretion of inhibitory molecules: arginase-1,reactive oxygen species, and immunosuppressive cytokines such as IL-10 and TGF-β. Importantly, malignant ascites contains a cytokine milieu that is highly enriched in pro-inflammatory, pro-angiogenic factors (IL-6, IL-8, CCL2), while the key mediators of anti-tumor immunity, IFN-γ, IL-2, and IL-12 are severely depleted. Thus, the cytokine imbalance directly promotes tumor cell proliferation. The epithelial-mesenchymal transition (EMT), neovascularization, and recruitment, retention, and functional polarization of TAMs and MDSCs are all coordinately regulated to establish a self-amplifying immunosuppressive loop (10). Therefore, therapeutic strategies that reprogram the key immune populations involved and disrupt their crosstalk with tumor cells and stromal components offer a potential approach to restore immune surveillance and achieve better clinical outcomes in malignant ascites. The therapeutic approaches discussed in this section range from preclinical proof-of-concept studies to early-phase clinical investigations.

One such strategy has been demonstrated in preclinical ovarian cancer models, where intraperitoneal administration of β-glucan combined with IFN-γ induced regression of peritoneal tumors and cleared malignant ascites. β-glucan alone sufficed to eliminate fluid-borne tumor cells through two nonredundant innate immune mechanisms: macrophage disappearance reaction-mediated intraperitoneal clotting and Dectin-1-Syk-dependent NETosis in the omentum. The addition of IFN-γ extended anti-tumor activity to solid peritoneal metastases via host T-cell immunity and IFN-γ receptor signaling. Mechanistically, this regimen expanded a distinct subset of immunostimulatory IL-27^+^ macrophages; neutralizing IL-27 *in vivo* impaired therapeutic efficacy, and IL-27/EBI3 co-expression in patient tumors correlated with improved overall survival. While these findings provide proof of concept in preclinical models for myeloid-activating immunotherapy in malignant ascites, the potent inflammatory nature of β-glucan and IFN-γ warrants careful toxicity monitoring in future clinical translation (40). Translational studies in human patients will be required to determine whether these preclinical observations are clinically relevant.

Evidence indicates that targeting the AURKA signaling axis can reprogram the ascites-associated immune microenvironment, because peritoneal metastases harbor tumor cells that are highly enriched for AURKA and secrete growth differentiation factor 15 (GDF15), both of which promote the expansion of CD11b^+^ PD-1^+^ TAMs that have defective phagocytic activity and fail to properly activate T cells. Intraperitoneal administration of the selective AURKA inhibitor MLN8237 efficiently depletes the immunosuppressive TAM subset, restores its antigen-presenting function, and therefore synergizes with anti-PD-1 immunotherapy to markedly enhance the cytotoxic activity of both CD8^+^ T cells and NK cells (41). CA125 (MUC16), abundantly expressed in malignant ascites, binds to Siglec-9 on NK cells and inhibits NK cell activation. The article describes the relation between CA125 and antibody-dependent cellular cytotoxicity (ADCC), and presents clear transcriptomic evidence that CA125 downregulates several NK cell activations-associated genes: NCR3 (encoding NKp30), NCR1 (encoding NKp46), GZMB (encoding granzyme B), and ITGB2 (encoding integrin β2), thereby impairing cytolytic function and weakening anti-tumor immunity. Hence, targeting the CA125/Siglec-9 interaction is a promising strategy. The strategy described restores NK cell effector activity and thus alleviates innate immune suppression in the ascitic microenvironment (42), while at the same time the VLA-4 (α4β1 integrin)/VCAM-1 axis mediates tumor cell adhesion to peritoneal mesothelial cells, promoting peritoneal metastasis and ascites accumulation. Preclinical studies have shown that blocking this adhesion pathway reduces intraperitoneal tumor dissemination (43).

Tumor cell-derived chemotherapeutic drug-loaded microparticles (drug-MPs) directly target TAMs and thereby efficiently induce their repolarization from the immunosuppressive M2 to the pro-inflammatory M1 phenotype. More importantly, mechanistic analysis has uncovered that after phagocytosis by macrophages, drug-MPs accumulate in lysosomes, where they activate lysosomal cytochrome P450 monooxygenases and the NOX2 complex, leading to robust intralysosomal reactive oxygen species (ROS) generation. This oxidative stress leads to lysosomal alkalinization (i.e., increased lysosomal pH), which causes Ca^2+^ efflux from lysosomes and thereby activates the downstream signaling cascades p38 MAPK, NF-κB, and TFEB, ultimately resulting in transcriptional reprogramming toward an M1-like, immunostimulatory state. After lysosome-to-nucleus translocation, the encapsulated chemotherapeutic agents engage the nuclear DNA sensor hnRNPA2B1, initiating the cGAS-STING pathway and therefore drives robust type I interferon (IFN-β) production, which in turn underlies a nicely orchestrated dual-action mechanism: innate immune activation via macrophage repolarization and adaptive immune priming via IFN-β-dependent dendritic cell maturation and CD8^+^ T-cell activation, together forming a synergistic anti-tumor immune response. This was demonstrated in a murine model of malignant ascites, where combination therapy with drug-MPs and anti-PD-1 antibody markedly extended overall survival compared to either agent alone, highlighting its potential to overcome concurrent suppression of both innate and adaptive immune checkpoints (44). These findings are based on a murine model and await clinical evaluation.

### 5.1 The liquid immune microenvironment

Beyond its role as a reservoir of immunosuppressive mediators, malignant ascites functions as a dynamic fluid-phase immune microenvironment—a “liquid immune microenvironment”—that connects primary tumors, peritoneal metastases, immune cells, stromal components, and soluble factors across anatomical sites (12). This fluid-phase ecosystem is not merely a passive recipient of shed tumor cells but an active compartment that shapes tumor progression, immune evasion, and therapeutic resistance through bidirectional crosstalk among its cellular and acellular constituents.

Single-cell transcriptomic profiling has revealed that ascites-enriched CD8^+^GZMK^+^ memory T cells share T cell receptors with tumor-infiltrating exhausted CD8^+^T cells in both primary and metastatic lesions, suggesting that the ascitic T-cell pool serves as a circulating reservoir for tumor-infiltrating lymphocytes (12). CD4^+^ Th1-like cells in ascites exhibit similar clonal relationships with their counterparts in tumor tissues, indicating dynamic T-cell trafficking between the fluid and solid compartments (12).

Tumor cells within ascites frequently form multicellular spheroids that survive in the peritoneal fluid, evade anoikis, and serve as seeds for peritoneal metastasis. These spheroids represent a chemoresistant and invasive cellular fraction that cannot be adequately modeled in conventional two-dimensional cultures, and they contribute to therapy resistance and metastatic dissemination (45). Macrophage ontogeny within the ascitic ecosystem exhibits marked compartment-specific heterogeneity: in ovarian cancer, tumor-enriched macrophages predominantly originate from circulating monocytes, whereas macrophages in ascites are largely of embryonic origin, suggesting distinct functional roles for macrophages in the fluid versus solid compartments (12).

The soluble phase of ascites contains a complex network of cytokines and chemokines that establish concentration gradients guiding immune cell trafficking. Chemokines such as CCL3, CCL4, CCL5, CXCL9, CXCL10, and CXCL11 recruit CXCR3^+^ Th1 cells into the peritoneal cavity, while elevated levels of immunosuppressive cytokines IL-10 and TGF-β counterbalance this recruitment, creating a microenvironment that paradoxically both attracts and functionally impairs effector T cells (10).

Important differences exist between ovarian and gastrointestinal cancer ascites. Ovarian cancer ascites is characterized by a higher proportion of tumor cells and a prominent role of spheroid-mediated metastasis (45). In contrast, colorectal cancer ascites contains a lower fraction of epithelial tumor cells and a higher proportion of T cells and macrophages. Gastric cancer ascites harbors a distinct epithelial-immune dual-phenotype cell population—validated by single-cell RNA sequencing and flow cytometry—that exhibits moderate malignant characteristics and potent immunoregulatory capacity, representing a potential immune evasion mechanism not prominently observed in ovarian cancer (46). Pancreatic cancer ascites is enriched for EMT-like tumor cells, consistent with the desmoplastic and aggressive behavior of metastatic pancreatic cancer, and this phenotype was associated with poorer response to oncolytic virotherapy in the H101 trial (13). In primary liver cancer, macrophage subsets in tumors have the potential to migrate to ascites (20), and ascitic microbiome profiles in HCC patients with portal vein tumor thrombosis correlate with systemic inflammatory markers (47).These cancer-type-specific differences in cellular composition and stromal-immune interactions have important implications for the rational design of tailored immunotherapeutic strategies. Consistent with this heterogeneity, gastric cancer was the only subgroup with a statistically significant OS benefit from catumaxomab (28), and patients with baseline relative lymphocyte count ≥ 13 % derived the greatest PuFS and OS benefit from M701 across gastric, colorectal, and ovarian cancer (32). Whether comparable predictive biomarkers apply to pancreatic or hepatobiliary ascites remains to be determined.

## 6 Other therapeutic strategies targeting immune microenvironment remodeling in malignant ascites

### 6.1 Anti-angiogenic agents

Bevacizumab is a humanized monoclonal antibody against VEGF that inhibits pathological angiogenesis and vascular hyperpermeability, thereby normalizing tumor vasculature, reducing ascites formation, alleviating intraperitoneal hypoxia, and decreasing local accumulation of immunosuppressive factors (e.g., VEGF, IL-10, TGF-β), thus partially reversing tumor-associated immune suppression. Kou et al. (48) carried out a single-arm phase I intraperitoneal (IP) dose-escalation trial that provided the first clinical evidence in favor of IP bevacizumab administration, and they established that no maximum tolerated dose (MTD) existed in the 2.5-7.5 mg/kg range, hence 7.5 mg/kg weekly was formally recommended as the phase II dose. This trial specifically enrolled patients with refractory malignant ascites due to advanced solid tumors. The regimen demonstrated favorable tolerability, with no grade ≥ 3 systemic adverse events (hypertension, proteinuria, gastrointestinal perforation) reported, and it achieved a disease control rate (DCR) of 61.5% (**Table 1**). Given the phase I dose-escalation design and the limited sample size (n=13), these findings should be interpreted primarily as safety and feasibility data to inform future trial design rather than as evidence of therapeutic efficacy.

### 6.2 Multi-kinase inhibitors

Preclinical evidence from a murine ovarian cancer model indicates that the multi-kinase inhibitor cabozantinib, which potently and simultaneously inhibits several receptor tyrosine kinases, including VEGFR-2 and MET, markedly suppressed ascites accumulation and reduced tumor burden. More importantly, cabozantinib treatment downregulated VEGFR-2 expression and profoundly enhanced systemic and intratumoral T-cell immunity: it increased CD3^+^ and CD8^+^ T-cell frequencies in the spleen, upregulated the early T-cell activation marker CD69 on T cells, boosted granzyme B production, and raised levels of immunostimulatory cytokines—IFN-γ, IL-2, CXCL10—in the TME. These findings indicate that cabozantinib’s anti-tumor activity is at least in part mediated by remodeling the immunosuppressive TME into one that is more immunoreactive (49). These observations derive from a murine model and require validation in human studies.

### 6.3 Targeting CD38 for anti-tumor synergistic therapy in malignant ascites

Evidence for CD38 targeting in malignant ascites is currently limited to case reports. Daratumumab is a human monoclonal antibody directed against CD38 that has anti-tumor activity mediated by ADCC, and there is now evidence supporting its efficacy in resolving malignant ascites. In primary effusion lymphoma (PEL), although tumor cells uniformly overexpress CD38, importantly daratumumab-mediated cytotoxicity in this setting is predominantly ADCC-driven rather than complement-dependent cytotoxicity (CDC). This resistance is attributed to constitutive high expression of complement regulatory proteins CD55 and CD59 on PEL cells. Of two patients with refractory PEL treated with daratumumab, the one with concurrent malignant ascites achieved complete ascites resolution and a substantial improvement in ECOG performance status. These findings highlight the therapeutic potential of CD38-targeted therapy not only for tumor control but also for the symptomatic management of malignant ascites in appropriate CD38-positive malignancies (50). However, this conclusion is based on a single patient with malignant ascites and requires confirmation in larger cohorts.

### 6.4 Plasma-oxidized saline synergistically reprograms the ascitic immune microenvironment

POS is a liquid rich in ROS (especially H_2_O_2_), obtained by treating physiological saline with cold atmospheric plasma. In a mouse model of Ehrlich ascites carcinoma, intraperitoneal administration of POS led to a reduction in tumor burden, promoted tumor cell apoptosis (as evidenced by increased caspase-3 activation), and increased lipid peroxidation in the ascitic supernatant. POS treatment reduced the total antioxidant capacity of tumor cells and moderately upregulated several cytokines in ascites: IL-6, IL-9, IL-10, and IFN-γ, thereby modulating the local immune microenvironment without causing appreciable systemic toxicity. More importantly, when combined with the chemotherapeutic agent oxaliplatin, POS synergistically enhanced anti-tumor efficacy while simultaneously attenuating the peritoneal inflammatory response caused by oxaliplatin alone (as shown by the decreased levels of IL-6 and IL-22). Thus, it represents a candidate for combination therapy (51). These findings are limited to a murine Ehrlich ascites carcinoma model and require further evaluation in clinically relevant models and human studies.

## 7 Scope, challenges, and perspectives

The present review focuses exclusively on the management of peritoneal carcinomatosis presenting with malignant ascites. This subgroup represents a clinically distinct population with prognostic and biological features that differ from those of peritoneal metastasis without ascites. The therapeutic strategies discussed—intraperitoneal immune checkpoint blockade, T-cell engagers, oncolytic viruses, and dendritic cell vaccines—are contextualized within the ascitic immune microenvironment. Solid-form peritoneal metastases without significant ascites are not within the scope of this review, as their biological behavior, immune landscape, and clinical management differ substantially from those of ascites-associated peritoneal carcinomatosis.

### 7.1 Challenges: toxicity and complex resistance mechanisms in combination therapy

Immunotherapy has great potential for the treatment of malignant ascites, but it is undeniable that clinical translation and widespread application are presently impeded by several important challenges, the most prominent being treatment-related toxicity management, especially in the context of combination regimens. Local delivery (e.g., intraperitoneal administration) can reduce systemic drug exposure, yet combining ICIs with TCEs may heighten the risk of CRS (32). Likewise, combining targeted agents with conventional chemotherapy can exacerbate local peritoneal inflammation. Therefore, careful dose titration, rational sequencing, and individualized scheduling are all essential to maximize efficacy while minimizing toxicity (48). Emerging myeloid-activating regimens, such as intraperitoneal β-glucan with IFN-γ, illustrate this challenge: both agents are potent inflammatory stimuli that may precipitate cytokine release syndrome or exacerbate peritoneal inflammation, underscoring the need for systematic safety evaluation before clinical application (40).

Resistance mechanisms can limit the long-term efficacy of combination immunotherapies, and there is now emerging evidence that expansion of SNCA-positive immune cell subsets—T cells, NK cells, myeloid cells—within the ascitic TME is strongly linked to resistance to anti-PD-1 ICB therapy (52). Therefore, SNCA appears not only as a novel predictive biomarker for ICB response but also as an excellent therapeutic target to overcome resistance. Furthermore, CD163 ^+^ TAMs promote EMT and acquisition of stem-like properties in tumor cells by delivering exosomal miR-221-3p, which downregulates ADAMTS6 and activates the AKT signaling pathway, driving acquired resistance to therapy (53).

### 7.2 Prospects for personalized treatment of malignant ascites

Because the etiological heterogeneity of malignant ascites is so clearly determined by primary tumor histotype, peritoneal metastatic burden, and host immune status, individualized therapeutic strategies are needed (4). Therefore, precision oncology for malignant ascites requires a paradigm shift toward biomarker-driven, patient-tailored interventions. A distinction should be made between prognostic and predictive biomarkers. Prognostic biomarkers are associated with clinical outcome irrespective of treatment, whereas predictive biomarkers identify differential benefit from a specific therapeutic intervention. In this review, PD-1^+^CD57^+^CD4^+^ T cells and CCR7^−^CD45RA^+^CD4^+^ naïve-like T cells are prognostic markers in ovarian cancer, as they correlate with overall survival and progression-free interval, respectively (54). In contrast, baseline relative lymphocyte count ≥13% is a candidate predictive biomarker for M701, as it identifies patients who derive greater PuFS and OS benefit from this T-cell engager (32). MAIT cells have been associated with chemotherapy response in ovarian cancer, but their ability to predict response to immunotherapy remains undetermined (12). IL-27/EBI3 co-expression in monocytes and macrophages correlates with improved overall survival and is therefore prognostic rather than predictive (40). An association between a biomarker and patient outcome does not establish its ability to predict response to a specific immunotherapy, and prospective validation is required before clinical use. Emerging evidence identifies specific immune cell subsets within the ascitic TME as robust, reproducible prognostic biomarkers. For example, PD-1^+^CD57^+^CD4^+^ T cells are linked to poor overall survival and acquired platinum resistance in ovarian cancer patients, whereas CCR7^−^CD45RA^+^CD4^+^ naïve-like T cells are strongly correlated with prolonged progression-free interval (54). Therefore, multiparametric flow cytometry-based immunophenotyping of ascitic leukocytes provides a foundation for developing predictive screening models that can rationally stratify patients for ICIs or TCEs. Complementary to immunophenotyping, specific immune signatures have emerged as candidate biomarkers from recent studies. MAIT cells in ascites have been associated with chemotherapy response in ovarian cancer, while IL-27/EBI3 co-expression in monocytes and macrophages correlated with improved overall survival in advanced ovarian cancer patients. These findings suggest that ascites-derived immune signatures may inform prognosis and treatment selection across different therapeutic modalities (12, 40).

Building on these observations, a biomarker-guided treatment decision framework can be proposed, although it remains investigational. In patients with PD-L1-high, T-cell-rich ascites, immune checkpoint blockade, particularly via the intraperitoneal route, may be considered to reinvigorate exhausted T cells (19, 20). In contrast, ascites enriched for TAMs and MDSCs may be more amenable to myeloid-reprogramming strategies, such as β-glucan combined with IFN-γ or agents targeting AURKA/GDF15 signaling, which aim to repolarize macrophages and restore antigen presentation (40, 41). A baseline peripheral blood relative lymphocyte count ≥13% has been associated with greater benefit from the T-cell engager M701, suggesting that lymphocyte abundance could serve as a predictive biomarker for TCE-based therapy (32). Other immune features, including MAIT cells and IL-27/EBI3 co-expression, have been linked to prognosis or chemotherapy response, but their role in predicting immunotherapy response requires prospective validation (12, 40). Integration of these biomarkers into prospective trials will be necessary to determine whether they can guide patient selection and improve outcomes.

The ascitic microbiome profile also holds promise as a novel biomarker, since recent evidence shows that Gammaproteobacteria and Acinetobacter are markedly enriched in ascites from HCC patients with portal vein tumor thrombosis (PVTT), and their relative abundances correlate positively with serum C-reactive protein (CRP) levels and inversely with apolipoprotein A-I (apoA-I) levels, suggesting a potential regulatory role of the microbiome-immune-metabolic axis in ascites pathogenesis and progression (47).

### 7.3 Development of *in vitro* models and predictive tools for ascites

Patient-derived organoid (PDO) technology provides a physiologically relevant *in vitro* model for mechanistic studies and drug sensitivity testing in malignant ascites. Li et al. (45) established the first gastric cancer-derived malignant ascites organoid (MADO) model and clearly demonstrated that organoids can be routinely cultured from ascitic tumor cells while preserving the histopathological architecture and genomic landscape, including mutational profiles and copy number alterations of key driver genes, of the original tumor. Drug sensitivity profiling using the MADO platform revealed marked inter-patient heterogeneity in responses to standard chemotherapeutic agents (oxaliplatin, 5-fluorouracil, paclitaxel). Thus, it has strong potential for individualized response prediction. Co-culturing MADOs with autologous peripheral blood-derived T cells effectively recapitulates tumor—immune interactions *in vitro*, therefore it is an ideal system for functionally evaluating the synergistic cytotoxicity of anti-PD-1 ICIs with chemotherapy, and hence provides a clinically translatable platform for optimizing personalized immunochemotherapy regimens (55). Moreover, integrating organoid-based pharmacosensitivity data with multi-omics profiling (genomics, transcriptomics, proteomics) offers a powerful approach to uncover the molecular basis of therapy resistance and design rational combination strategies.

Because of the increasing need to integrate multidimensional data, decision-support systems based on multimodal machine learning are becoming increasingly important for improving predictive accuracy. A recent paper presented an application of random survival forests to jointly model clinical parameters and ascites-derived immune features, obtaining a C-index substantially higher than that of unimodal models for predicting overall survival. Moreover, SHAP (Shapley Additive Explanations) analysis provided an interpretable, quantitative assessment of the direction and magnitude of each immune subset’s contribution to prognostic risk (54). Most importantly, it highlights that these integrative models are ideal for dynamically tracking therapeutic resistance signals and therefore aid clinicians in optimizing drug dosing while managing toxicity, especially in combination therapy scenarios.

### 7.4 Comparative assessment of therapeutic strategies for malignant ascites

The strategies discussed in this review differ in efficacy, safety, limitations, and clinical maturity. All clinical studies discussed in this comparative assessment specifically enrolled patients with malignant ascites. Catumaxomab and M701 are the only agents supported by randomized controlled trials. In the pivotal phase II/III trial, catumaxomab plus paracentesis prolonged median PuFS (46 vs. 11 days; HR = 0.254; *p* < 0.0001) and median TTPu (77 vs. 13 days; HR = 0.169; *p* < 0.0001), with an OS benefit confined to the gastric cancer subgroup (71 vs. 44 days; *p* = 0.0313) (28). CRS occurred in approximately 72% of treated patients, mostly grade 1-2, with life-threatening SIRS in 0.6%; HAMA formation was associated with improved outcomes (31). In the randomized phase II trial of M701, median PuFS was 75 vs. 25 days (HR = 0.43; *p* = 0.0065) and median OS was 110 vs. 76 days (HR = 0.68; *p* = 0.1443); patients with baseline RLC ≥13% had median PuFS 125 days and median OS 152 vs. 76 days (*p* = 0.0155) (32). CRS occurred in 4% of M701-treated patients, all grade 1-2, and SAE incidence was comparable to paracentesis alone. Direct head-to-head comparisons are lacking.

Intraperitoneal immune checkpoint inhibitors and oncolytic viruses are supported by early-phase single-arm trials. Nivolumab (n=9) produced a clinical response rate of 77.8% with only grade 1 puncture-site tenderness (19). Sintilimab (n=21) achieved an objective ascites response rate of 43.75%, median OS 17.6 weeks, and grade 3 AEs in 33.3% of patients, with no grade ≥4 AEs (20). H101 (n=25) achieved median TTRP 45 days, with grade 3 treatment-related AEs in 8% of patients and no grade 4 treatment-related AEs (13). These studies are limited by single-arm design and small sample size.

Dendritic cell vaccines and CAR-T cells are supported by case reports only. A dendritic cell vaccine induced antigen-specific CD8^+^ T-cell responses and reduced ascites tumor cells in one patient (17). A CAR-T case report described ascites reduction and CAR-T cell detection in ascites (34). Tumor-associated lymphocytes combined with chemotherapy were evaluated in a non-randomized prospective study of 32 patients, with an objective ascites response rate of 90.9% versus 37.5% for TALs alone and 53.8% for chemotherapy alone; the study was limited by non-randomized design and single-center conduct (37). TAM/myeloid-targeting strategies, ferroptosis-inducing hydrogels, and plasma-oxidized saline remain preclinical (40, 41, 44, 51).

In summary, catumaxomab remains the only agent with randomized phase III evidence, and M701 the only agent with randomized phase II evidence. Intraperitoneal immune checkpoint inhibitors and oncolytic viruses are supported by early-phase single-arm trials. Cellular therapies and myeloid-targeting strategies remain at earlier stages of development. Safety profiles differ, with CRS and HAMA formation characteristic of T-cell engagers, while immune checkpoint inhibitors and oncolytic viruses have shown more favorable local tolerability in early-phase trials.

## 8 Conclusion

Malignant ascites remains a challenging clinical condition with limited treatment options, and palliative fluid management through paracentesis continues to be the cornerstone of care for most patients. However, growing insights into the immunobiology of the ascitic microenvironment have begun to translate into mechanism-informed therapeutic strategies.

Among these, T-cell engagers have the strongest clinical rationale for localized immunotherapy. Catumaxomab, supported by randomized phase III evidence, has demonstrated consistent benefit in prolonging puncture-free survival, though its overall survival effect is limited to the gastric cancer subgroup and the drug has undergone regulatory withdrawal and reauthorization. The emerging bispecific antibody M701 has shown encouraging results in a phase II randomized controlled study, with a phase III trial currently ongoing.

Intraperitoneal immune checkpoint inhibitors and oncolytic viruses are conceptually attractive and supported by early-phase clinical data, but their role remains investigational. The available evidence consists primarily of small, single-arm studies, and confirmatory trials are needed before these approaches can be recommended in routine practice.

Myeloid- and TAM-targeting strategies are biologically compelling and have shown activity in preclinical models, but they have not yet progressed to clinical evaluation in malignant ascites. Their translational potential, while promising, requires formal testing in human studies.

Looking further ahead, personalized approaches based on patient-derived ascites organoids for drug sensitivity testing and multimodal machine learning models for predictive stratification offer a roadmap for precision immunotherapy. However, these tools remain at an early stage of development and require prospective validation before integration into clinical decision-making.

It should be noted, however, that the evidence base for most strategies discussed herein remains early-stage: among intraperitoneal immunotherapies for malignant ascites, only catumaxomab has been evaluated in a randomized phase III trial (subsequently withdrawn from the market and re-approved in 2025), while the majority of other approaches are supported by phase I/II single-arm studies, case reports, or preclinical models. Given this evidence profile, our review does not aim to endorse specific agents but rather to provide a mechanistic framework.

In summary, the field of immunomodulatory therapy for malignant ascites is evolving from palliation toward mechanism-based intervention. While catumaxomab and other T-cell engagers provide a clinical foundation, most other approaches remain at various stages of early development, and rigorous clinical evaluation will be essential to define their ultimate role in patient care.

## Glossary

BiTE: Bispecific T-cell engager, A class of T-cell engagers consisting of two single-chain variable fragments (scFvs) linked in tandem, designed to bridge T cells and tumor cells for targeted cytotoxicity
Catumaxomab: A trifunctional, bispecific antibody targeting EpCAM and CD3, the first T-cell engager approved for intraperitoneal treatment of malignant ascites
CRS: Cytokine release syndrome, A systemic inflammatory response characterized by elevated cytokines (e.g., IL-6, IFN-γ) following T-cell activation; can occur with T-cell engagers and immune checkpoint inhibitors
EpCAM: Epithelial cell adhesion molecule, A transmembrane glycoprotein frequently overexpressed in epithelial tumors and widely used as a target for bispecific antibodies in malignant ascites therapy
HAMA: Human anti-mouse antibody, An immune response elicited by murine-derived therapeutic antibodies; in catumaxomab-treated patients, HAMA positivity has been associated with improved clinical outcomes
ICIs: Immune checkpoint inhibitors, Monoclonal antibodies that block inhibitory immune checkpoints (e.g., PD-1, PD-L1, CTLA-4) to restore T-cell-mediated anti-tumor immunity
ICD: Immunogenic cell death. A form of cell death that releases damage-associated molecular patterns (DAMPs), tumor-associated antigens (TAAs), and pro-inflammatory cytokines, thereby activating adaptive anti-tumor immunity
*In situ* vaccination: A therapeutic strategy that induces tumor-specific immune responses at the tumor site through local delivery of agents (e.g., oncolytic viruses, immune modulators) that trigger immunogenic cell death and activation of antigen-presenting cells
Malignant ascites: An abnormal accumulation of fluid in the peritoneal cavity resulting from peritoneal carcinomatosis or advanced intra-abdominal malignancies, associated with poor prognosis, immunosuppression, and impaired quality of life
MDSCs: Myeloid-derived suppressor cells, A heterogeneous population of immature myeloid cells that suppress T-cell activation and effector functions through various mechanisms, including arginase-1, reactive oxygen species, and immunosuppressive cytokines
OVs: Oncolytic viruses, Genetically engineered or naturally occurring viruses that selectively replicate in and lyse tumor cells while inducing immunogenic cell death and remodeling the tumor immune microenvironment
PDOs: Patient-derived organoids, Three-dimensional *in vitro* cultures derived from patient tumor tissues or ascitic cells that recapitulate histopathological and genomic features of the original tumor, enabling drug sensitivity testing and personalized therapy modelling
PD-1/PD-L1 axis: A key immune checkpoint pathway in which programmed cell death protein 1 (PD-1) on T cells engages its ligand PD-L1 on tumor cells or antigen-presenting cells, leading to T-cell exhaustion and immune suppression
Peritoneal cavity: An anatomically and immunologically distinct compartment harboring a resident immune cell repertoire (e.g., macrophages, T cells, B cells) organized into milky spots; serves as a critical site for antigen presentation and local immune surveillance in peritoneal malignancies
Tregs: Regulatory T cells. A subset of CD4^+^ T cells that suppress immune responses and maintain immune homeostasis; in the tumor microenvironment, they contribute to immune evasion and resistance to immunotherapy
TCEs: T-cell engagers, Bispecific antibodies that simultaneously bind a tumor-associated antigen (e.g., EpCAM) and the CD3 component of the T-cell receptor complex, inducing MHC-unrestricted T-cell activation and tumor cell lysis
TAMS: Tumor-associated macrophages, Macrophages infiltrating the tumor microenvironment that often exhibit an M2-like immunosuppressive phenotype, promoting tumor progression, angiogenesis, and immune evasion
